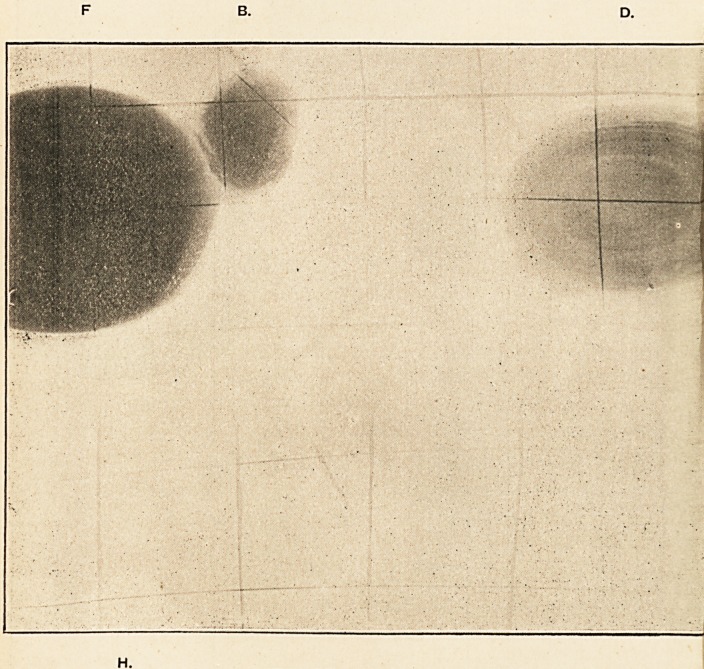# The Effect of the Röntgen Rays on Calculi

**Published:** 1897-03

**Authors:** James Swain

**Affiliations:** Assistant-Surgeon, Bristol Royal Infirmary


The Bristol

Medico-Chirurgical Journal.

MARCH, 1897.

### THE EFFECT OF THE RÖNTGEN RAYS ON CALCULI.

### WITH THE REPORT OF A CASE OF RENAL CALCULUS IN WHICH THE DIAGNOSIS WAS CONFIRMED BY SKIAGRAPHY.

BY

JAMES SWAIN, M.S., M.D. Lond., F.R.C.S. Eng.,
*Assistant-Surgeon, Bristol Royal Infirmary.*

THE great difficulty of distinguishing nephro-lithiasis from many other pathological conditions of the genito-urinary tract—especially early tubercular disease of the kidney—is well known; but, except in the case reported below, the Röntgen rays have not yet proved of much practical value in the diagnosis of renal calculus.[1]

It can be, however, only a matter of time; and there is little doubt that a more extended knowledge of the methods now employed will enable us to graphically prove the existence of calculi not only in the kidney, but also in the urinary bladder,[2] gall-bladder, and intestine. Meanwhile, it may be profitable to consider the behaviour of calculi outside the body when exposed to the X rays, as Mr. Morris[3] has already done to some extent.

[1] *Lancet*, 1896, ii. 1631. [2] *Ibid.*, 1897, i. 169. [3] *Ibid.*, 1896, ii. 1367.

2

For this purpose a series of calculi of different composition has been taken, and their general appearance and size are well shown photographically in Plate I. As nearly as possible their thickness was uniform, about $\frac{3}{8}$ of an inch, in order that they might show more clearly the relative effect of exposure to the X rays for an equal number of minutes. The coil as used gave a minimum spark of three inches, and the focus tube was placed at a distance of six inches from the sensitive plate, and as nearly as possible directly over its centre.

In Plate II. we see the relative permeability of these calculi to the X rays after an exposure of four minutes. For purposes of comparison, some silver coins were placed on the sensitive plate, and as these are practically impermeable to the rays, we may regard the shadows of the coins as the standard of absolute impenetrability. It will be noticed that the deepest shadow is given by the two oxalate of lime calculi (B and F), which shows by comparison with the shadow of the coins that they may be regarded as almost impermeable to the rays—the coin beneath the larger calculus giving only a slightly deeper shadow than the calculus itself. The shadow given by the phosphatic calculus (D) is not so deep, and the coin beneath can be more plainly seen. Next in order comes the uric acid calculus (E); but this is much more easily penetrated by the rays than the former calculi, and the shadow of the coin beneath it stands out very strongly. Lastly, we get the still fainter shadow caused by the biliary calculus (C), which shows that its power of intercepting the X rays is comparatively slight. It follows from this, that the probability of diagnosis by these means is greatest in the oxalate of lime calculus, and least in the biliary calculus—phosphatic and uric acid calculi occupying an intermediate position.

It is interesting in this connection to consider Röntgen's[1] statement that, generally speaking, the denser the object, the deeper the shadow; in other words, the transmissibility of the X rays varies in an inverse ratio with the specific gravity of the body. This, however, is not the only factor in determining

[1] *Sitzungsb. d. phys.-med. Gesellsch. zu Würzb.*, 1895; translated in *Nature*, 1896, liii. 274, and reprinted in a Special Issue of the *Photogram*, Feb. 1896.

PLATE I.

*Photograph of Calculi—approximately of natural size.*

---

**A.** Biliary Calculus, similar to **C.**

**B.** Oxalate of Lime Calculus (Renal).

**C.** Biliary Calculus containing about 90 per cent. of Cholesterin.

**D.** Section of a Phosphatic Calculus (Vesical). A few streaks of Uric Acid can be seen.

**E.** Section of a Uric Acid Calculus (Vesical).

**F.** Section of an Oxalate of Lime Calculus (Vesical).

**D.** **E.** **F.**

the effect of the rays on calculi, and is not borne out in every case, as will be seen. By weighing the calculi in air and water respectively, I ascertained their specific gravities as accurately as possible, and found them as follows:—The larger oxalate of lime calculus (F), 1.924; the smaller oxalate of lime calculus (B), 1.780; the uric acid calculus (E), 1.669; the phosphatic calculus (D), 1.555; and the biliary calculus (C), 1.024. Arranged in the order of the highest specific gravity, the greatest permeability to the X rays and the greatest density of the shadow, we find that they do not entirely agree with the law enunciated, thus:—

| | Specific Gravity. | Permeability. | Density of Shadow. |
|---|---|---|---|
| 1. | Oxalate of lime. | Biliary. | Oxalate of lime. |
| 2. | Uric acid. | Uric acid. | Phosphatic. |
| 3. | Phosphatic. | Phosphatic. | Uric acid. |
| 4. | Biliary. | Oxalate of lime. | Biliary. |

Lime salts appear to hinder the transmission of the rays to a considerable extent, and it may be due to their presence in the phosphatic calculus that we get a deeper shadow than in the case of the uric acid calculus, in spite of the latter having a higher specific gravity than the former. The opacity of bone is due to a similar cause (see below).

A series of plates was prepared to show the effect of "time exposures" of the rays on the calculi. The sensitive plates were crossed by intersecting portions of silver wire for purposes of comparison as regards impenetrability, and the radiographic centre in each case was vertically over the point of intersection of the central wires. In addition to the calculi, a piece of human kidney covered with part of a rib was placed on the sensitive plate. The Plates (III., IV.) illustrating the effects found practically speak for themselves. In Plate III. after an exposure of eight minutes we find the shadows much less distinct than in Plate II., which was exposed for four minutes. Indeed, with exposures of one minute, two minutes, four minutes (Plate II.), eight minutes (Plate III.), and sixteen minutes (Plate IV.), the shadows were found to become gradually fainter, so that in the last Plate (IV.) only the oxalic acid and phosphatic calculi, and the merest trace of a rib are

PLATE II.

*To show the comparative transparency of the calculi shown in Plate I. after an exposure to the X rays of four minutes. Four coins were placed on the plate to give an idea of absolute density.*

**B.** Oxalate of Lime Calculus.
**C.** Biliary Calculus.
**D.** Phosphatic Calculus.
**E.** Uric Acid Calculus.
**F.** Oxalate of Lime Calculus.

seen. The edges of the calculi being thinner than their central portions, the shadow at the periphery is less dense: but it may be noted that the shadow at the end nearer the radiographic centre is less dense than that more remote; showing that, other things being equal, the penetrating power of the rays is increased by proximity to the object—a result which we should naturally expect. As a matter of fact, an exposure of eight minutes at twelve inches, gave practically the same depth of shadow as an exposure of two minutes at six inches. In other words the effect of the X rays, as in the case of ordinary light, varies inversely as the square of the distance of the object. It is noticeable in Plate IV. that the smaller oxalic acid calculus (B), which had a lower specific gravity than the larger one (F), gives a less dense shadow (*v. supra*). The lesson conveyed by this experiment is that, in attempting to diagnose calculi by the X rays, a short exposure may give a better result than a long one—a fact discovered in the case referred to later. What we have to find out is the amount of exposure necessary for the rays to penetrate the ordinary tissues without completely penetrating the calculus. If this time be exceeded, we may miss some calculi altogether, as in Plate IV.

On looking at Plate III. it is evident that the shadow caused by a uric acid calculus is less deep than that given by a piece of rib—especially if the relative thickness is taken into consideration—and much less dense than that given by a rib and portion of kidney superimposed. The diagnosis, therefore, of uric acid calculi in the kidney is likely to be a matter of some difficulty; and it follows that calculi in the gall-bladder, which give a lighter shadow still, will be even more difficult.

It can be seen in Plate III. that the osseous portion of the rib gives a deeper shadow than the costal cartilage attached; due no doubt, as suggested by Morris,[1] to the lime salts contained in the bone.

In the above experiments the focus tube was placed at a distance of six inches from the sensitive plates, and the same "developer" (pyrogallic acid) was used in each case. Each negative became "denser" as the exposure was prolonged. It

[1] *Loc. cit.*

PLATE III. (Reduced).

*The effect of the X rays after an exposure of eight minutes.*

**A.** Biliary Calculus.
**B.** Oxalate of Lime Calculus.
**C.** Biliary Calculus.
**D.** Phosphatic Calculus.
**E.** Uric Acid Calculus.
**F.** Oxalate of Lime Calculus.
**G.** Portion of human Kidney.
**H.** Piece of human Rib.

is important, however, to distinguish between the effects of a prolonged exposure on the negative and on the print, *e.g.* although some of the calculi have disappeared in the print of Plate IV., the negative itself showed them all by transmitted light. Speaking generally, it was found that with a more prolonged exposure the detail of the more transparent objects

F B. D.

H.

PLATE IV. (Reduced).

*The effect of the X rays after an exposure of sixteen minutes.*

B. Oxalate of Lime Calculus. F. Oxalate of Lime Calculus.
D. Phosphatic Calculus. H. Rib.

became more marked both in negative and print; but at length the negatives, although showing more detail to the eye, became so dense as to be unprintable. This difference between the negative and print was not observable in the plates taken of the living subject,—all these negatives being "thin."

It is, however, desirable to see the negatives in all cases to avoid missing shadows not evident in the print. A new kind of paper has just been introduced which permits of the skiagram being taken on it directly, and so dispensing with the need of an ordinary negative—an advance in skiagraphy which will remove the source of error referred to.

The case in which a skiagram of a renal calculus was obtained in the living subject, previous to operation, was kindly sent to me on August 15th, 1896, by Mr. T. F. Edgeworth, of Cotham. The history is briefly as follows:—

A. C., a rather pallid and spare man, aged 27 years, had suffered for ten years from "colicky pains" in the left side of the abdomen, with the occasional passage of blood in the urine for the first two years. At the beginning of his illness the attacks of pain were frequent, lasting for some hours; but eight years ago they became much less frequent, and continued so until two years ago, since which time the pains have been of almost daily occurrence, and have entirely prevented him from following his occupation of a tailor's cutter. There had been no blood in the urine for the past eight years, except in May, 1896, when he had a transient hæmaturia after running with otter hounds, but he has known that the urine contained albumen "for many years." The attacks of pain commence and end suddenly, and feel like "windy spasms" in the neighbourhood of the left kidney, and are often relieved by the passage of wind "upwards or downwards." The pains go across the abdomen chiefly, but also somewhat towards the pubes and left groin, but he has never noticed any retraction of the testis. Vomiting has occurred once only. "Jolting" does not produce pain.

Skiagrams showed a distinct shadow in the region of the left kidney, but the negatives were too "thin" to permit of a print being satisfactorily reproduced. No enlargement of the kidney could be felt, but examination caused an aching pain in the left loin. The bladder was sounded for stone, but none felt. His weight was 112 lbs. The urine was amber-coloured, clear, specific gravity 1033, slightly acid, and gave a fairly thick precipitate of albumen on boiling. Microscopically, scattered red blood-corpuscles and pus-cells were seen. All over the field were numerous small octahedral crystals of oxalate of lime. No urinary casts were seen, but here and there were large round granular cells which were thought to be degenerated epithelial cells of renal origin.

On September 3rd, 1896, chloroform was administered, and I made an oblique incision in the left loin. The kidney was found to be small and deeply placed, and on the posterior surface at the hilum a hard rounded body could be felt in the pelvis of the kidney. To facilitate manipulation, a piece of silk was temporarily passed through the kidney in order to hold it up to the surface. To avoid going through the pelvis of the kidney (which is often followed by a urinary fistula), and to save hemorrhage from incising a great deal of renal tissue, an incision was made through the parenchyma of the kidney on the posterior surface near the hilum. Hemorrhage was rather free, but was checked by the insertion of the finger into the wound. The stone, which was about half as large as a walnut was removed with the finger, and a periosteal elevator used as a scoop The wound was drained, and dressed with carbolic sponge-cloths.

At first there was a good deal of hemorrhagic discharge throug[illegible] the wound, and the urine passed *per urethram* was of a brigh[illegible] red colour. The temperature never rose above 100.6° F. an[illegible] urine ceased to come through the wound after the second da[illegible] No crystals were found in the urine after operation, all traces [illegible] blood soon disappeared, and the patient made a rapid convalescenc[illegible] On November 21st, 1896, he reported himself as feeling perfect[illegible] well, as entirely free from pain, and he had gained a stone and a ha[illegible] in weight.

Although this case was regarded as one of nephro-lithiasis there were several symptoms that tended to throw doubt o[illegible] the correctness of the diagnosis. The patient suffered severel[illegible] from flatulent dyspepsia, and stated that medicine containin[illegible] bismuth almost always relieved his pain. Symptoms of dyspepsi[illegible] are common enough in renal calculus, and can be explained b[illegible] the transference of nervous impulses through the connection o[illegible] the pneumogastric nerve with the renal plexus; but we do no[illegible] find as a rule that the actual attacks of renal colic are relieve[illegible] by such simple measures as a dose of bismuth. Then again h[illegible] stated that attacks were induced by keeping quiet indoors, an[illegible] could to some extent be warded off by exercise in the fresh air[illegible] no retraction of the testis had ever been noticed; vomiting ha[illegible] occurred only once; and a diet of cucumber or green vegetabl[illegible] (which are known to contain oxalates) would be certain [illegible] induce an attack. Such symptoms suggested the possibili[illegible] of the case being one of simple oxaluria (without calculu[illegible] engrafted on chronic Bright's disease. Under these circum[illegible] stances the shadow found, after the patient had been ski[illegible] graphed, was of great value in corroborating the diagnosis [illegible] renal calculus. The shadow given after an exposure of thirt[illegible] five minutes was not very deep, but with an exposure of twen[illegible] minutes the presence of the calculus was made abundant[illegible] clear. The importance of a short exposure was subsequent[illegible] borne out by experiment, and has already been referred t[illegible] The skiagrams were taken with a coil capable of giving [illegible] spark of nineteen and a half inches, but which was worked [illegible] about half its full strength. A slight dermatitis of the abdom[illegible] was set up by the exposure to the X rays,—the focus tu[illegible]

having been placed about twelve inches from the body,—but this disappeared in a few days.

The calculus itself, of which a photograph is shown in Plate I. (B), was of great interest, being one of those rare varieties known as the "white" or "crystalline" oxalate of lime calculus. It weighed 148 grains, and consisted of almost pure oxalate of lime. In shape it was roughly triangular, with mammillated angles, and measured in inches $1\frac{1}{8} \times \frac{7}{8} \times \frac{5}{8}$. The whole surface was studded with large well-formed octahedral crystals, which were easily visible to the unaided eye, and reflected the light over the greater part of the calculus as if it had been studded with broken glass. Two of the angles of the calculus were of a brownish colour, apparently from contact with the mucous membrane which had been made to bleed, but the greater portion of it was greyish white, where it had been washed by the urine excreted, and the crystals freely deposited without staining from any accompanying hemorrhage. Although it is not extremely rare to find a few octahedral crystals on some of the smaller "mulberry" calculi, there appear to be very few examples of such an extensive deposit of crystals as in the specimen referred to. Dr. Perkins,[1] of Ogden, U.S.A., removed a similar one, weighing 82 grains; Lionel Beale[2] says he possessed one which was obtained from a horse, and others are referred to by Prout[3] and Hutchinson.[4]

It will be inferred from what has been said that the skiagraphic detection of this calculus was largely due to its composition, although the patient was below the ordinary age for an oxalate of lime calculus, which belongs to middle life. It is, however, remarkable that oxalate of lime calculi are more common in Bristol and its neighbourhood than in other parts of England. In the classification of calculi made by Richard Smith,[5]

[1] *Ann. Surg.*, 1896, xxiv. 204.

[2] *Urinary and Renal Derangements and Calculous Disorders*, 1885, p. 236.

[3] *On the Nature and Treatment of Stomach and Renal Diseases*, 5th ed. 1848, p. 575.

[4] *Atlas of Illustrations of Pathology*, New Syd. Soc., 1888, fasc. vi.

[5] *A Statistical Inquiry into the Frequency of Stone in the Bladder in Great Britain and Ireland*, 1820.

of the Bristol Royal Infirmary, the frequency of this form[illegible] calculus is evident. Prout[1] tells us that nearly 50 per ce[illegible] of the Bristol calculi contain oxalate of lime, and that ab[illegible] 16 per cent. of them consist of the salt nearly pure; where[illegible] according to Beale,[2] only 5.12 per cent. of the calculi[illegible] the College of Surgeons; 9.13 per cent. at Guy's Hospit[illegible] and 3.16 per cent. in the Norwich collection; are composed [illegible] pure oxalate of lime. The reason for this undue proportion [illegible] oxalate of lime calculi in this district is difficult to account [illegible]

It is a popular fallacy to suppose that the Bristol water [illegible] responsible for the occurrence of oxalate of lime calculi. L[illegible] other "hard" waters, it contains a considerable quantity [illegible] carbonate and sulphate of lime; but calcium is ingested w[illegible] many articles of food, and in some of these (rhubarb, cabba[illegible] celery, potatoes, bread, cocoa, tea, coffee, etc.) it exists [illegible] combination with oxalic acid. No trace, however, of oxa[illegible] acid is to be found in Bristol or any other potable water, and [illegible] presence in the human economy can best be accounted for by [illegible] imperfect katabolism of the disintegrated elements of albumino[illegible] tissues or of an excessive supply of proteid material.[3]

Notwithstanding the qualities which have been attribu[illegible] to them,[4] it is difficult to assume that Bristolians were so mu[illegible]

[1] *An Inquiry into the Nature and Treatment of Diabetes, Calculus, and* ot[illegible] *Affections of the Urinary Organs*, 2nd ed. 1825, p. 102. [2] *Op. cit.*, p. 246.

[3] According to M'Kendrick (*A Text Book of Physiology*, 1888, vol. i. p. 1[illegible] oxalic acid, apart from its ingestion in food stuffs, in combination with li[illegible] may arise from the incomplete oxidation of uric acid ($C_5H_4N_4O_3$) into [illegible] oxalic acid ($C_3H_2O_5$) which then becomes converted into oxalic acid, thu[illegible]

Mesoxalic Acid. Oxalic Acid.

$$C_3H_2O_5 + O = CO_2 + C_2H_2O_4$$

These views are combated by Dr. Dunlop in an article on "[illegible] Excretion of Oxalic Acid in Urine, and its Bearing on the Patholo[illegible] Condition known as Oxaluria," *J. Path. & Bacteriol.*, 1896, iii. 389.

[4] Byron, in *English Bards and Scotch Reviewers*, ed. 1809, says:—

"Too much in turtle Bristol's sons delight;
Too much o'er bowls of rack prolong the night:
If Commerce fills the purse, she clogs the brain,
And Amos Cottle strikes the lyre in vain."

Chatterton's disappointment with his fellow-citizens led him to write *Last Verses*:—

"Farewell, Bristolia's dingy piles of brick,
Lovers of Mammon, worshippers of Trick!

"Farewell, ye guzzling aldermanic fools,
By nature fitted for Corruption's tools!"

e self-indulgent than their fellows, as to account for their ggerated liability to the "mulberry" calculus.

I have to acknowledge my indebtedness to Dr. Cotton of stol—an indefatigable worker in skiagraphy—for many uable suggestions, and for the excellent plates (I. to IV.) h which this paper is illustrated. The skiagrams which e sent to me with the patient had been previously prepared University College, under the direction of Prof. Chattock, at suggestion of Mr. Edgeworth.

## Figures and Tables

**PLATE I. f1:**
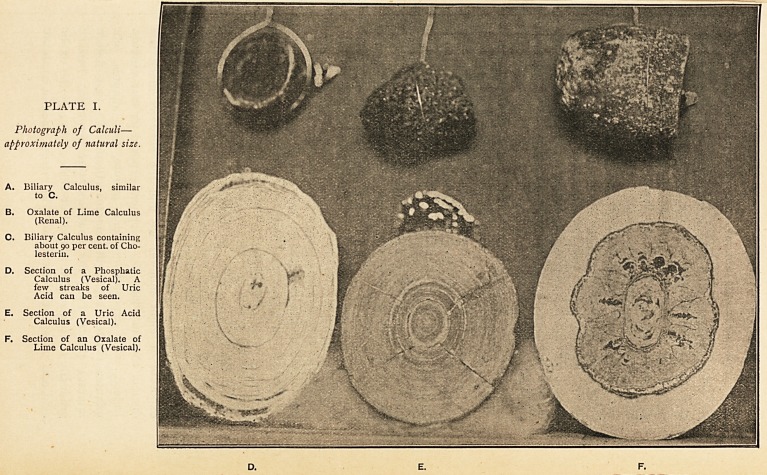


**PLATE II. B. E. F. f2:**
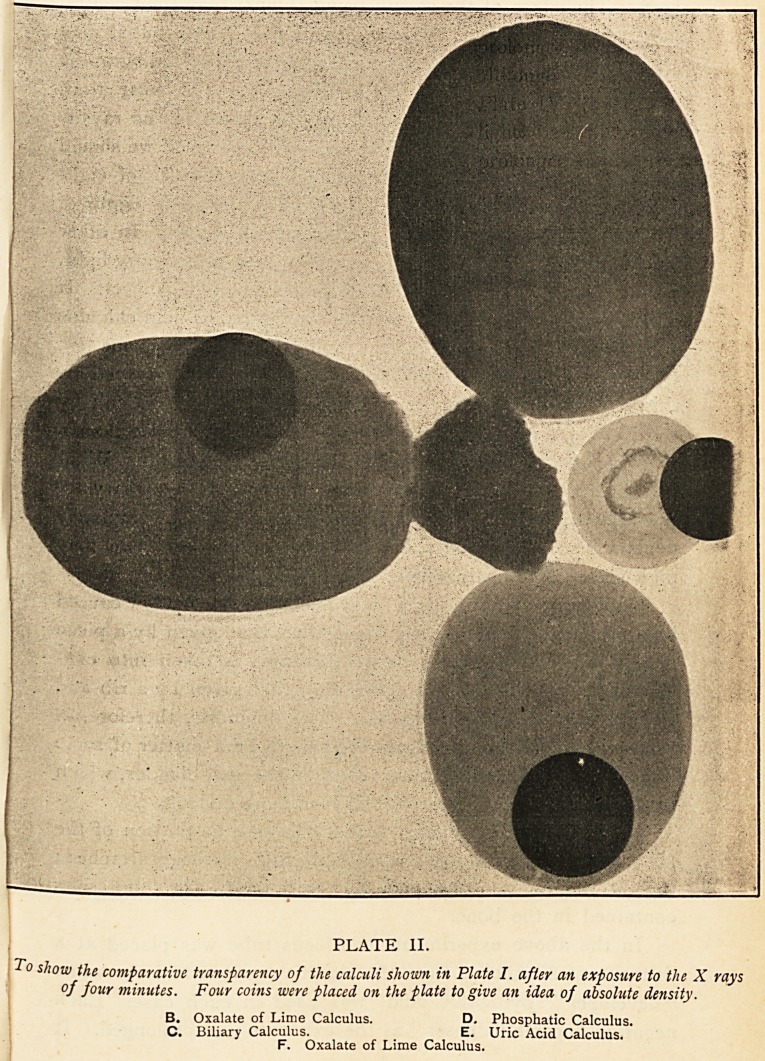


**PLATE III. A. E. f3:**
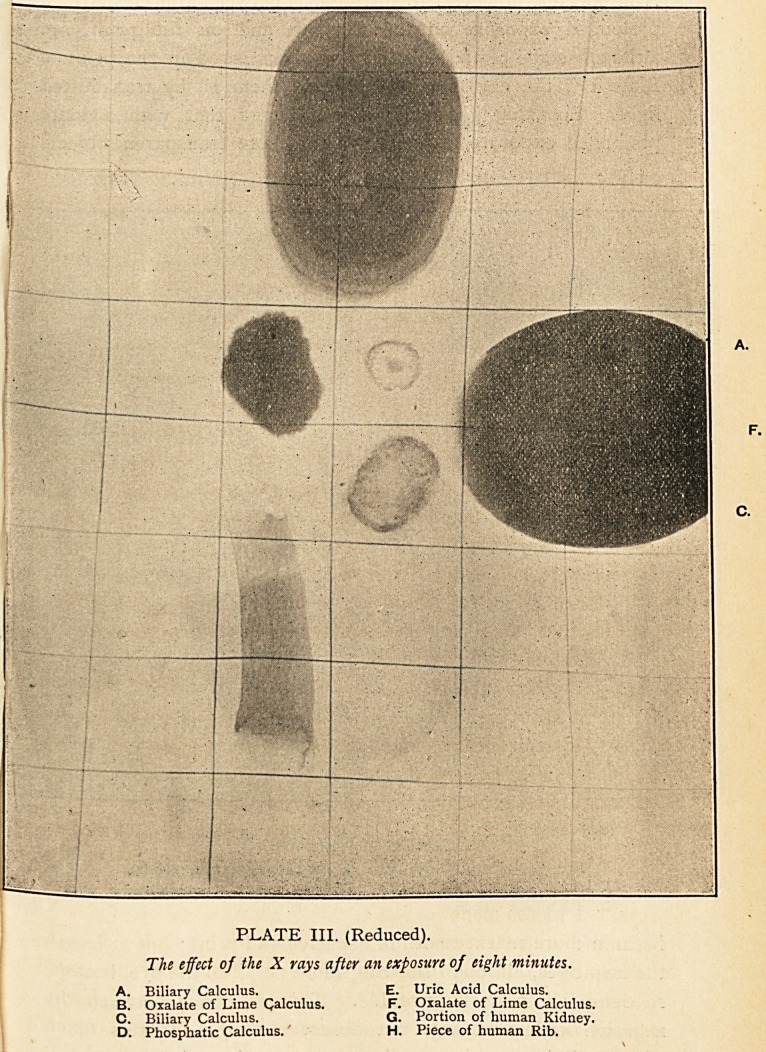


**PLATE IV. B. F. f4:**